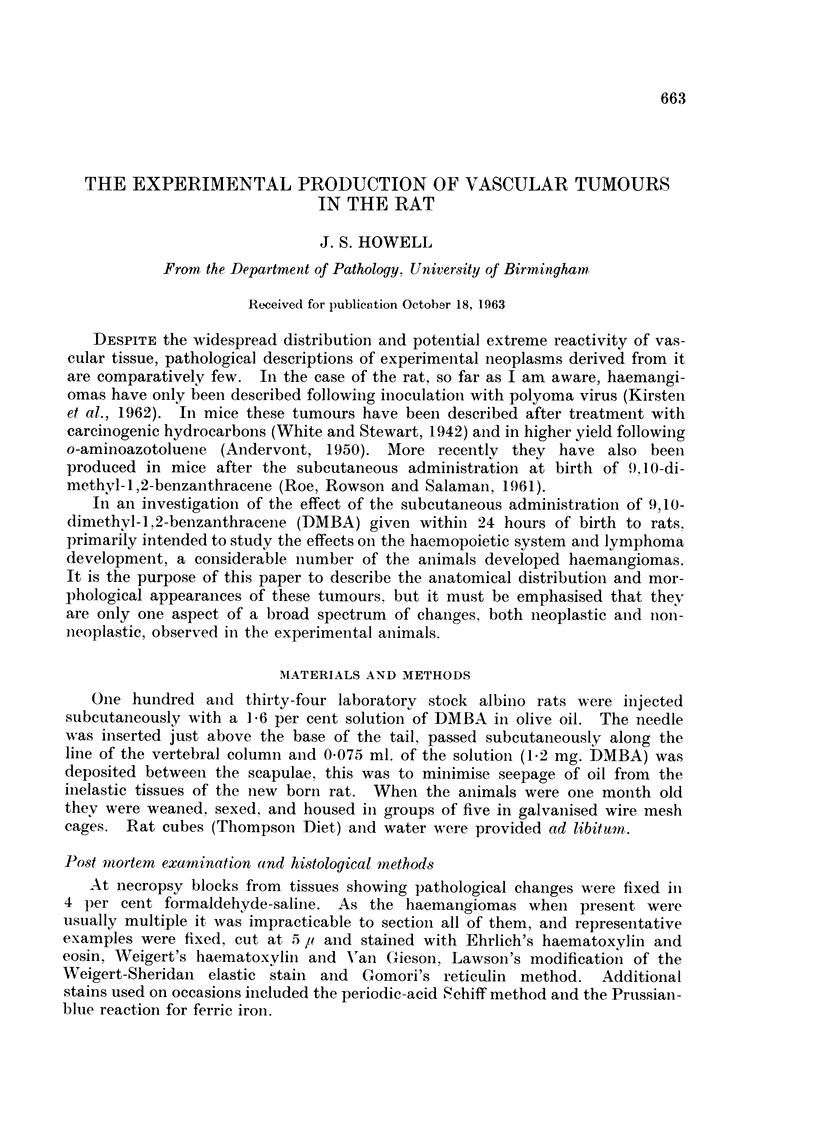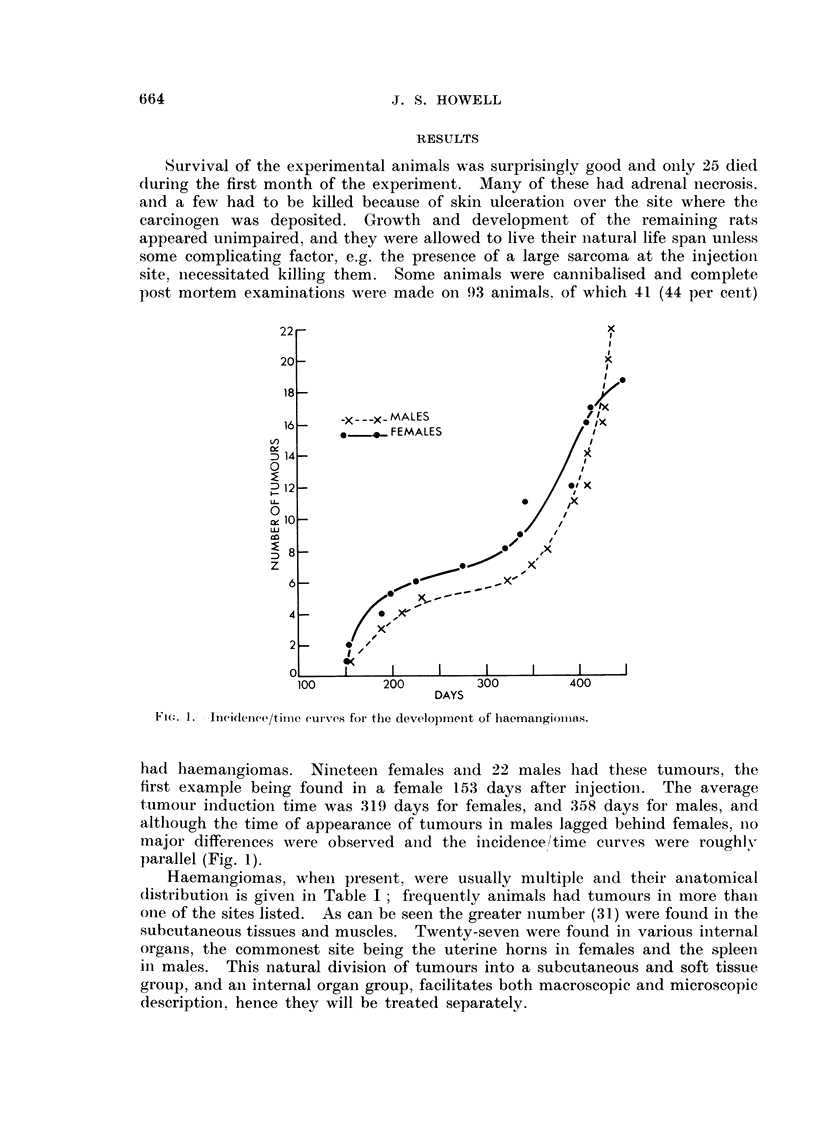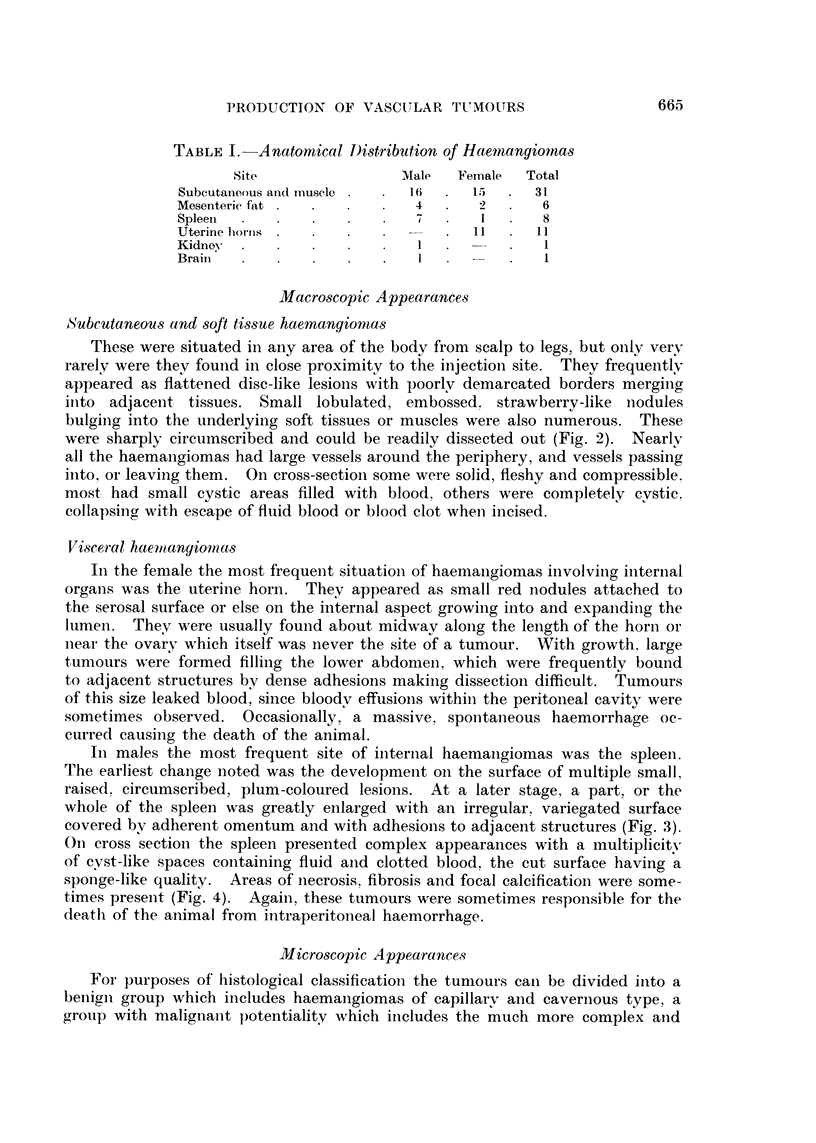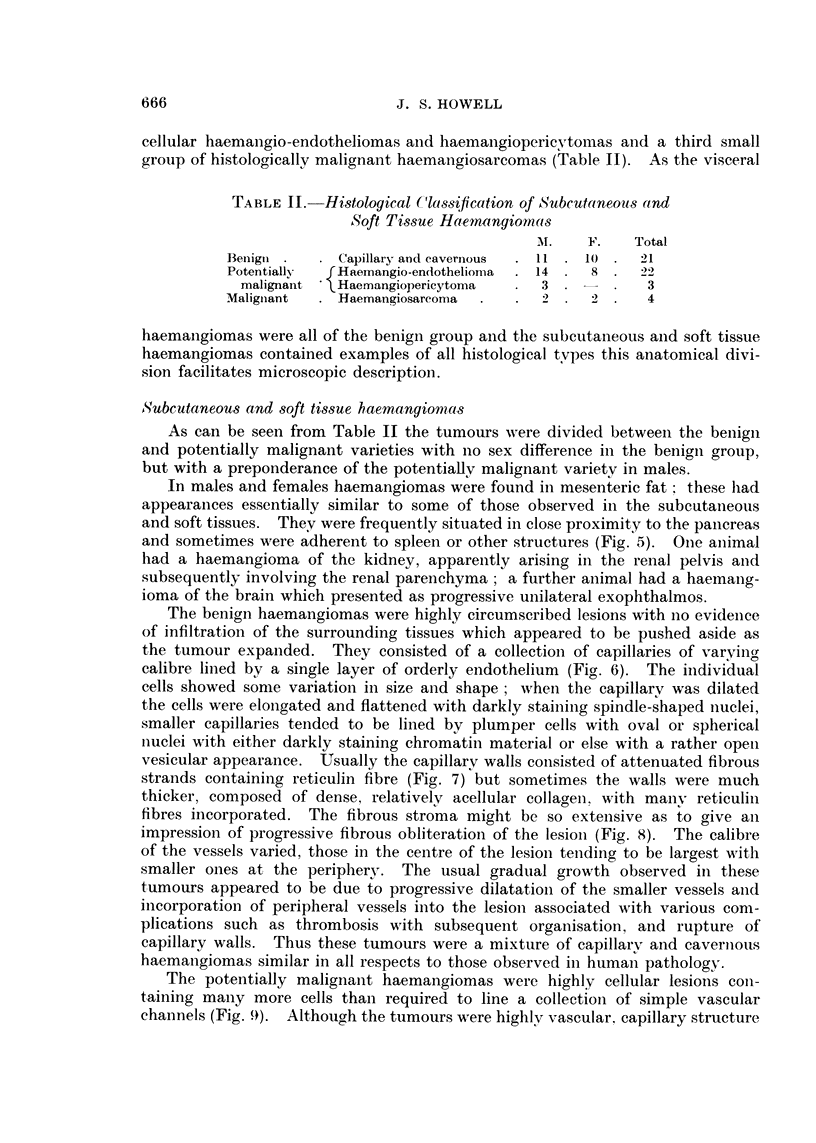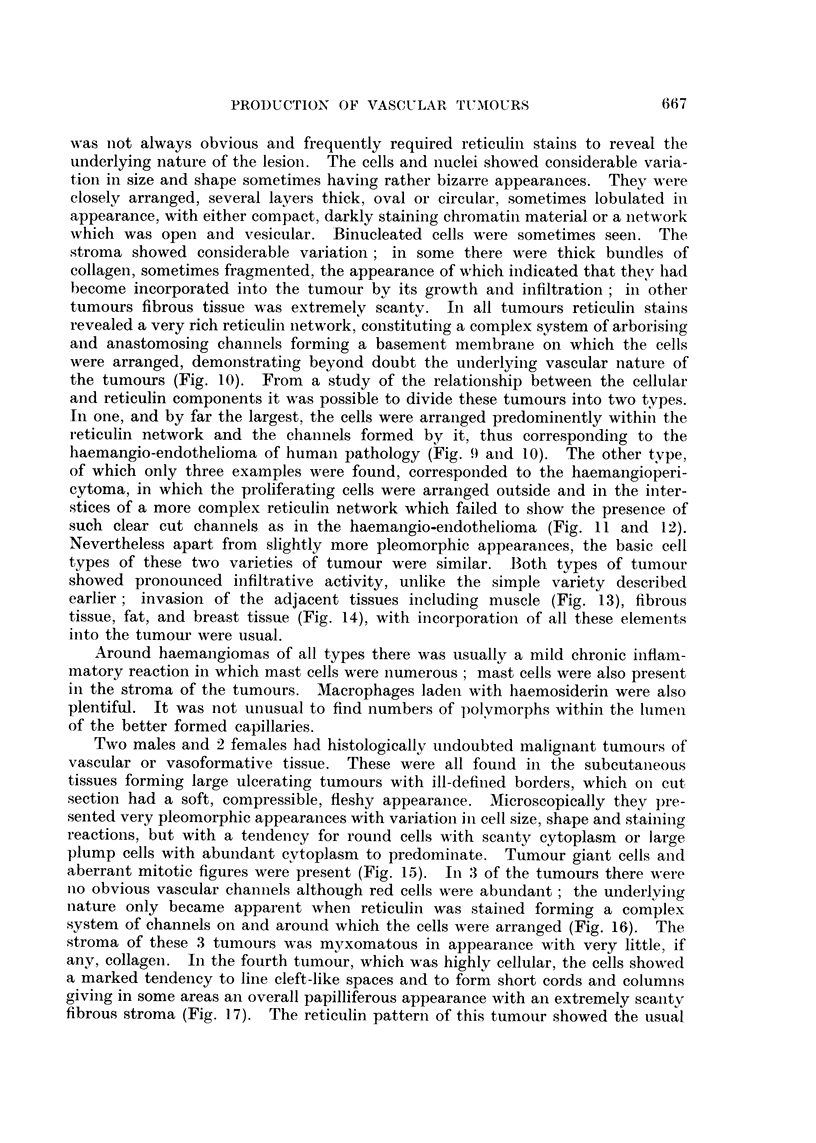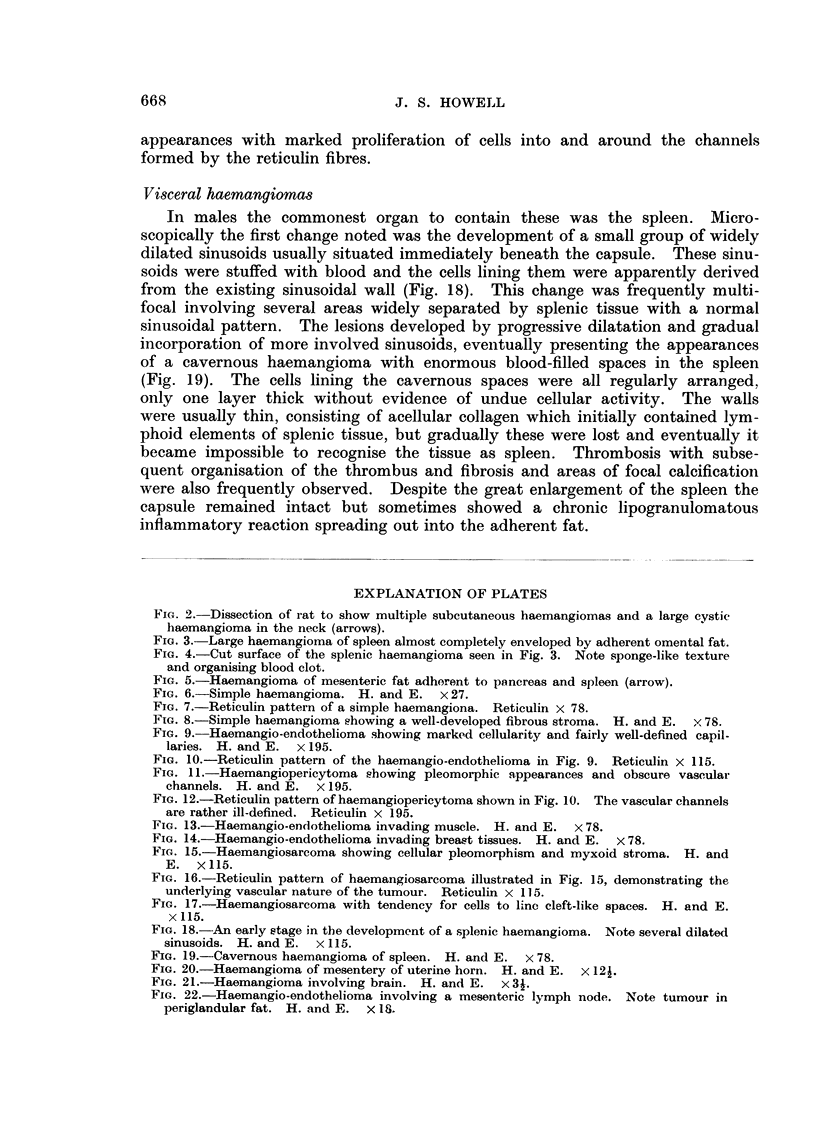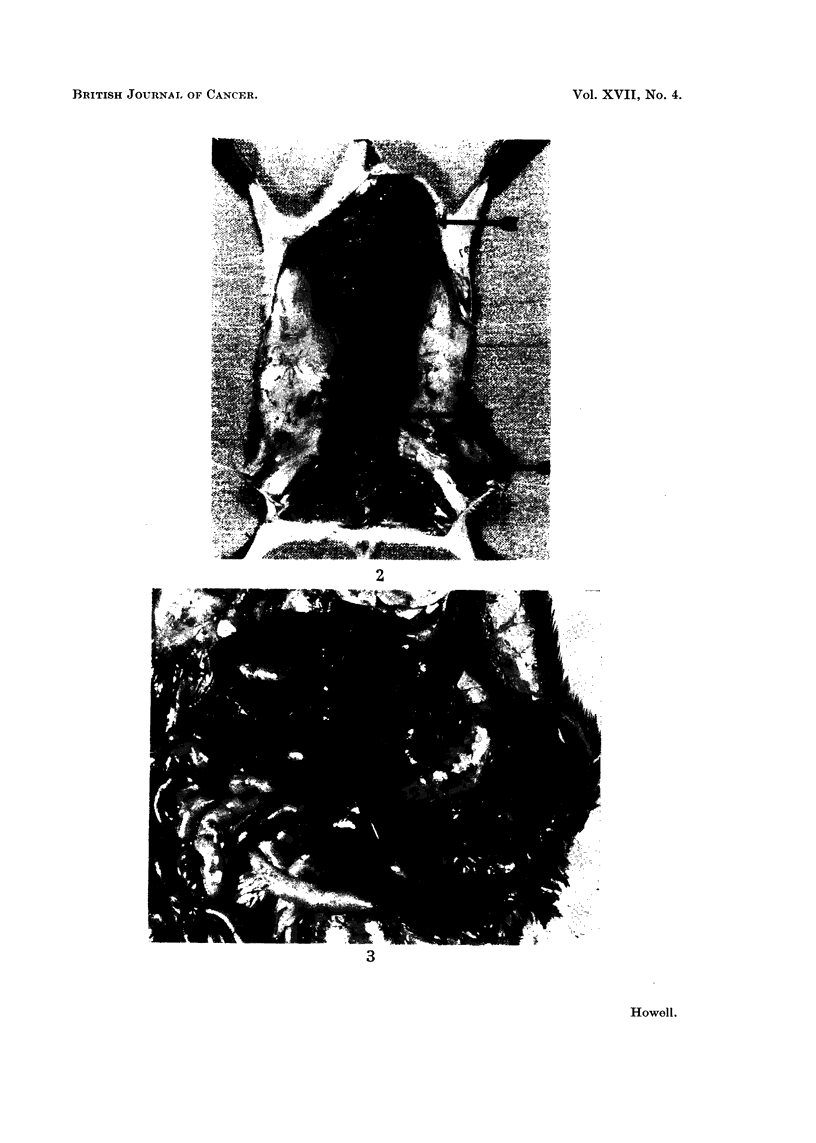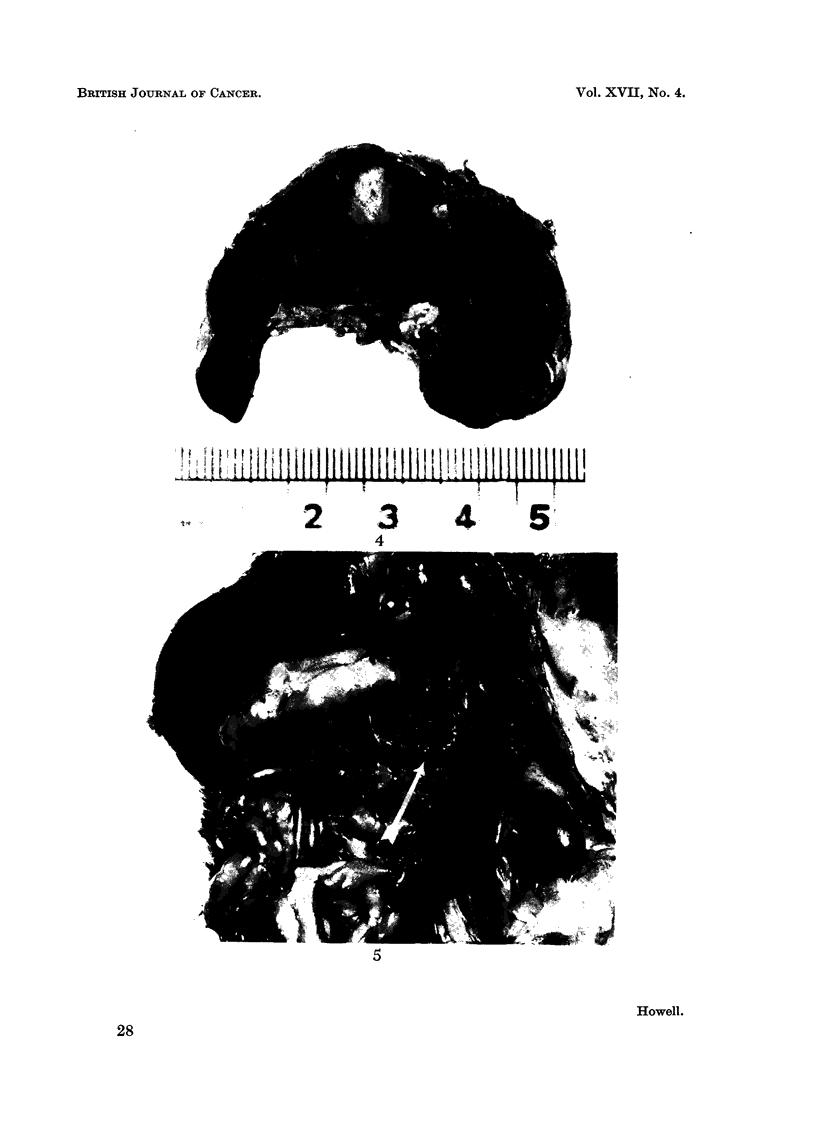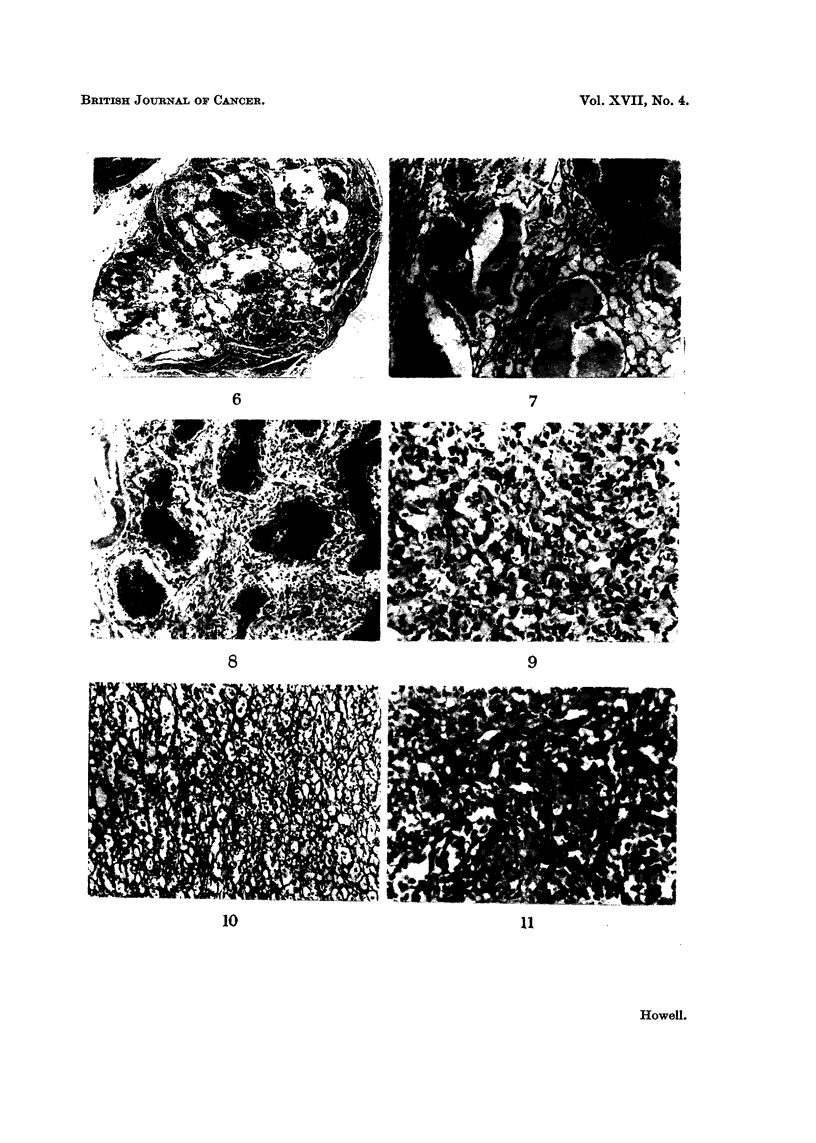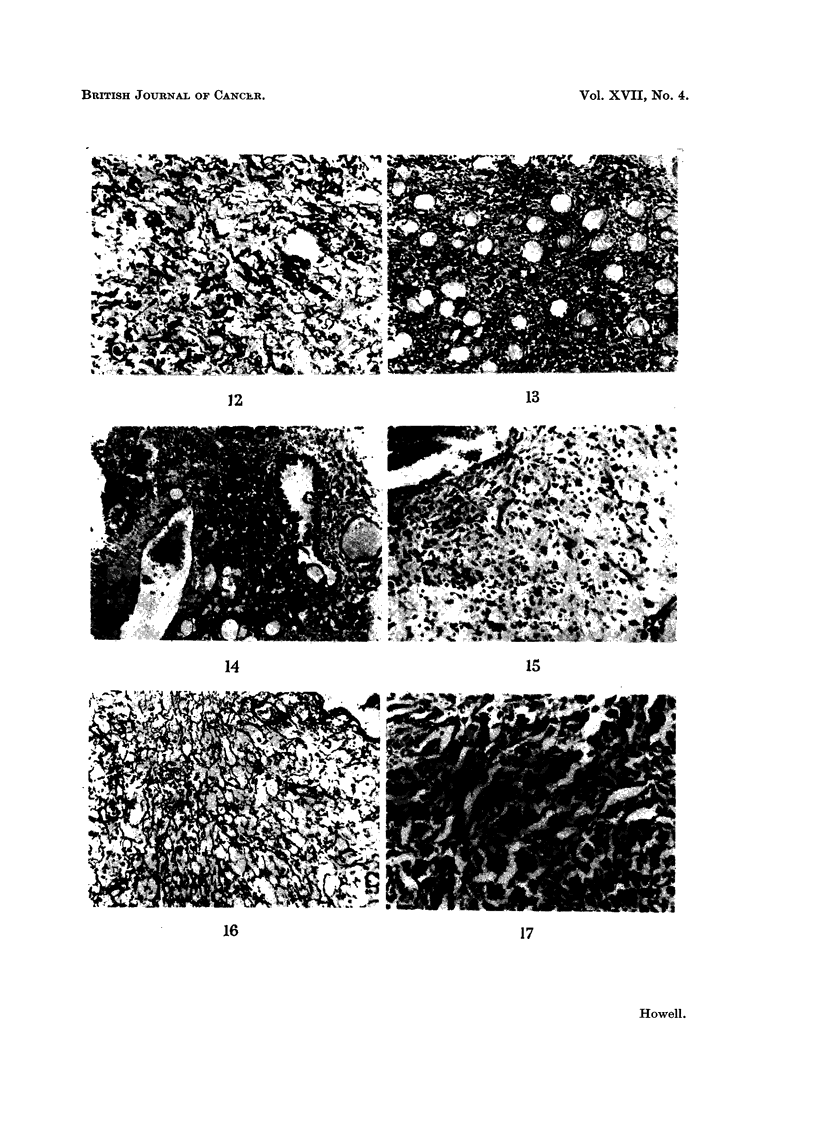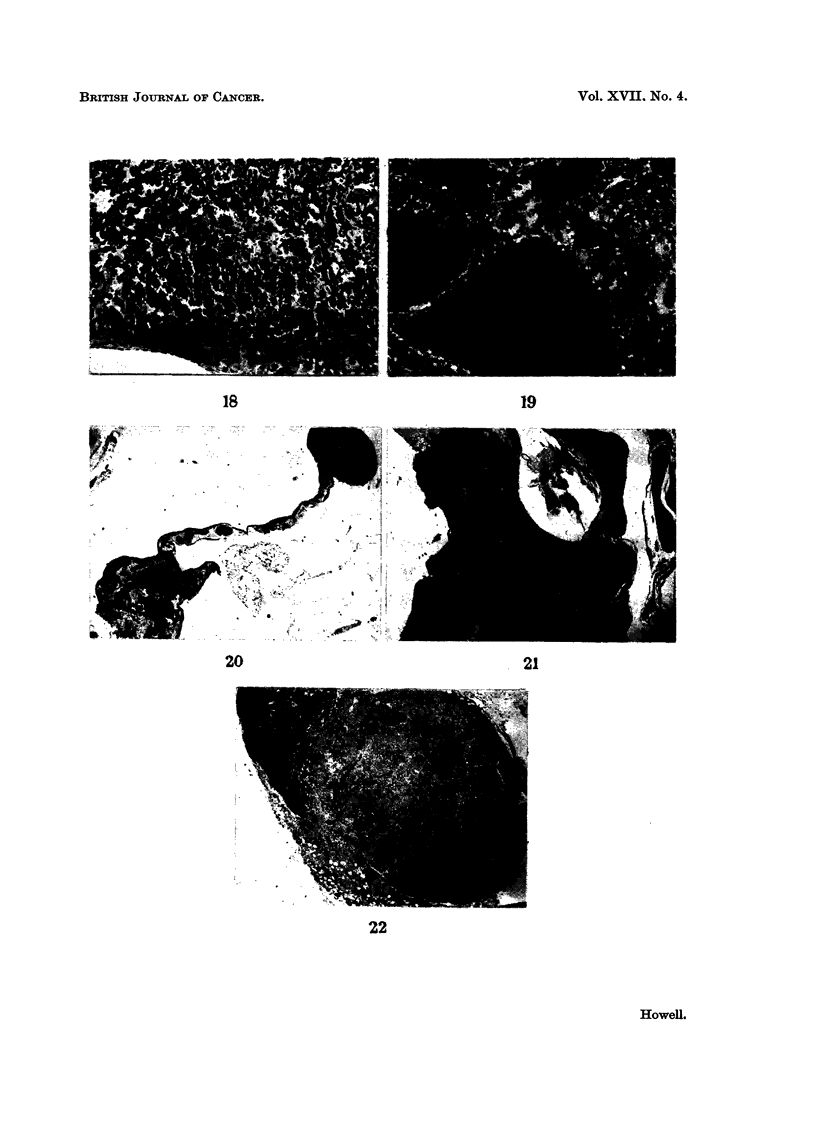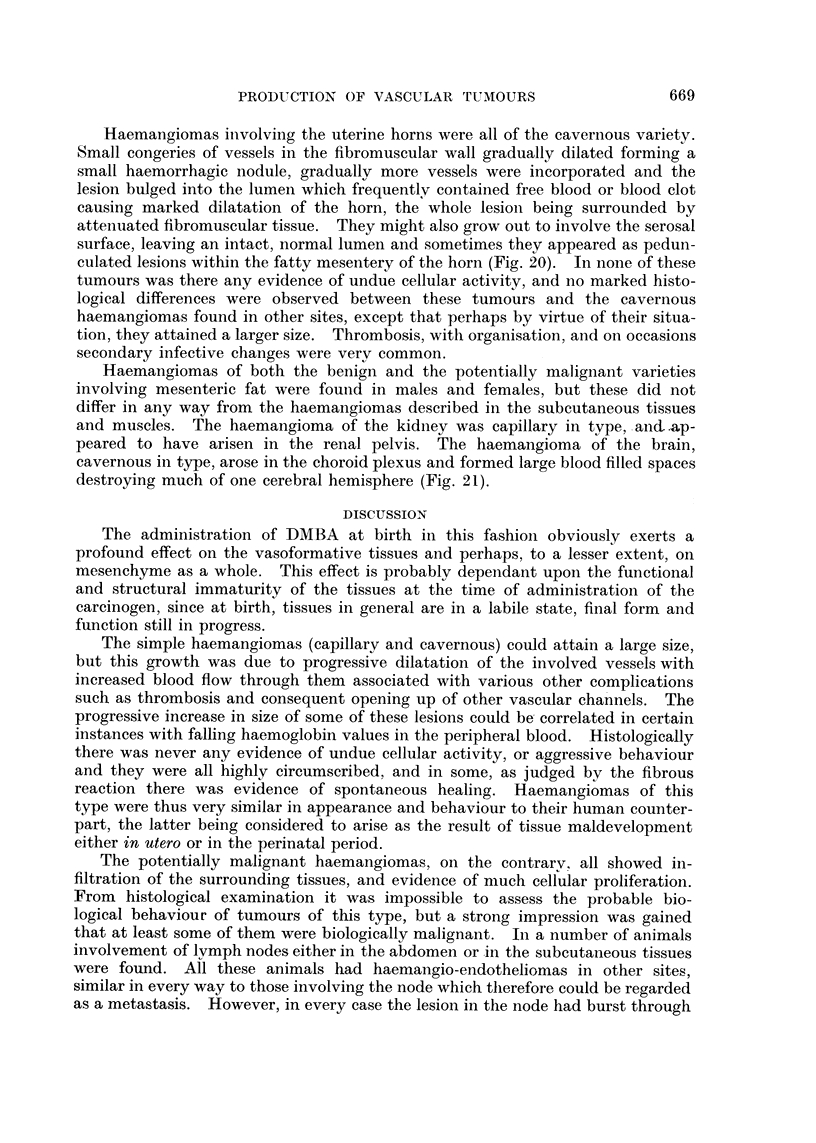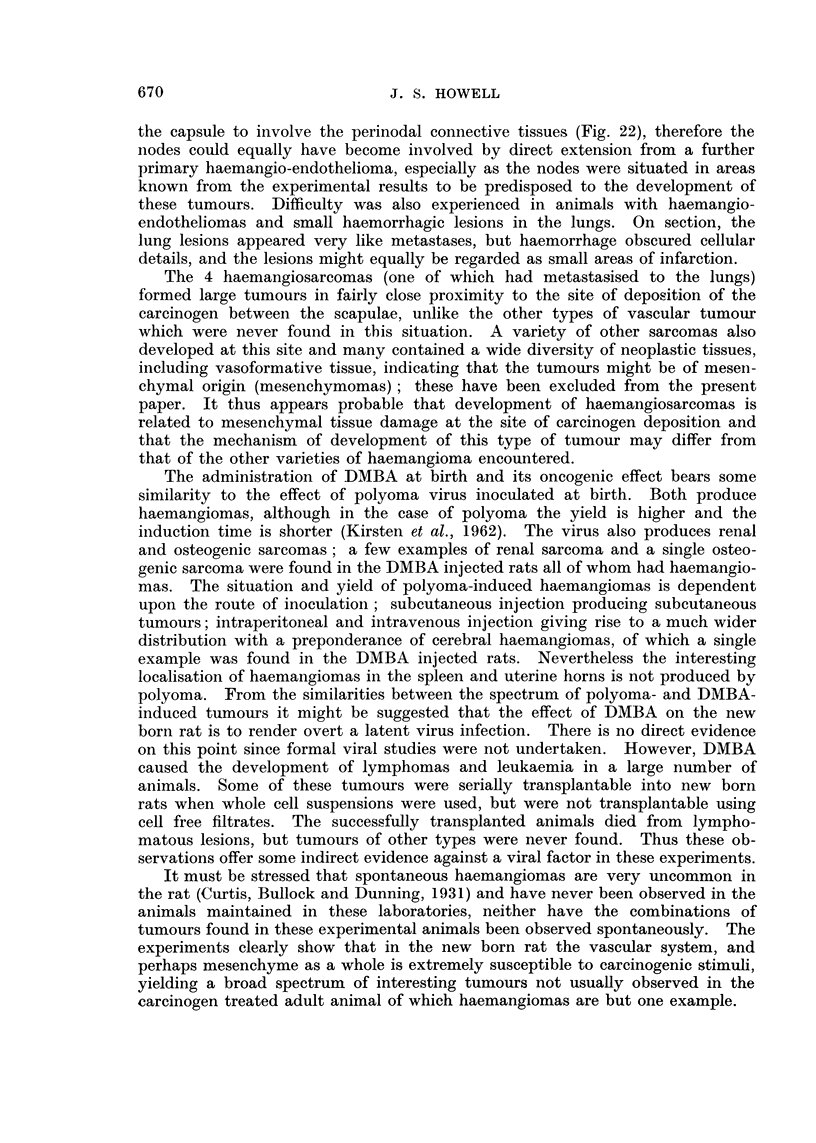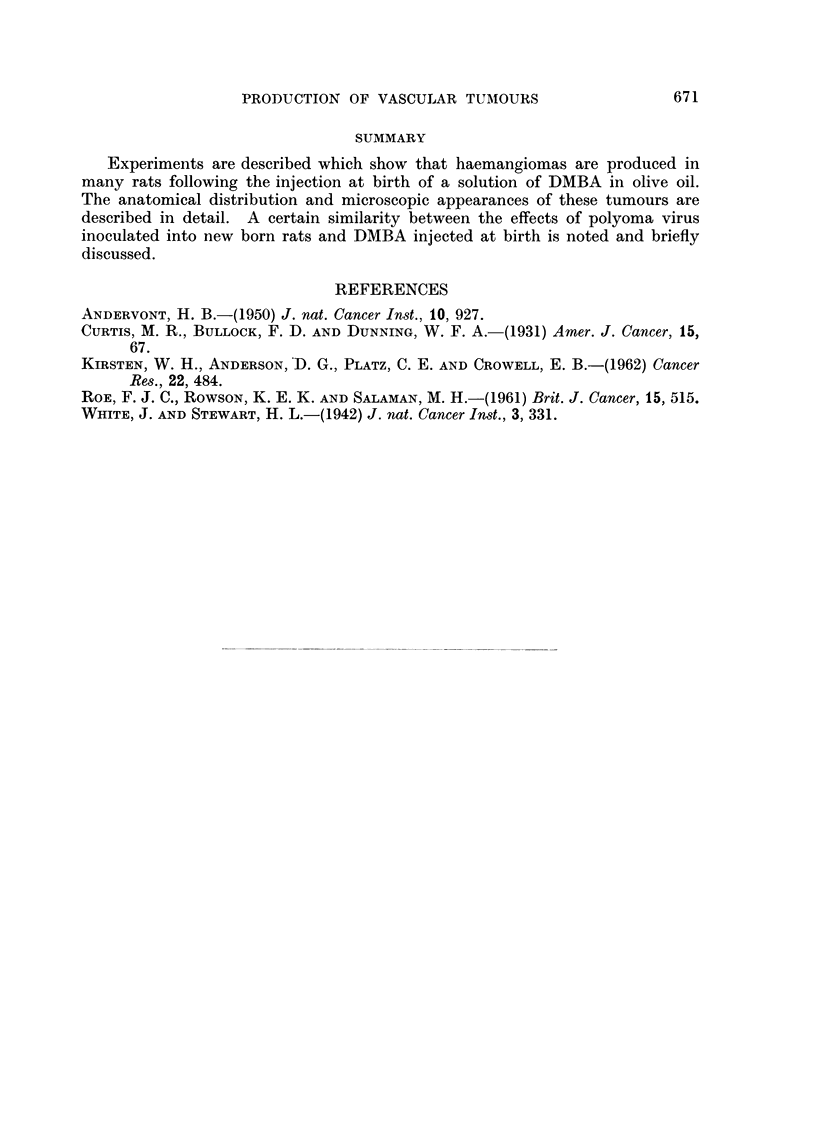# The Experimental Production of Vascular Tumours in the Rat

**DOI:** 10.1038/bjc.1963.84

**Published:** 1963-12

**Authors:** J. S. Howell

## Abstract

**Images:**


### THE EXPERIMENTAL PRODUCTION OF VASCULAR TUMOURS IN THE RAT

J. S. HOWELL

*From the Department of Pathology, University of Birmingham*

Received for publication October 18, 1963

DESPITE the widespread distribution and potential extreme reactivity of vascular tissue, pathological descriptions of experimental neoplasms derived from it are comparatively few. In the case of the rat, so far as I am aware, haemangiomas have only been described following inoculation with polyoma virus (Kirsten *et al.*, 1962). In mice these tumours have been described after treatment with carcinogenic hydrocarbons (White and Stewart, 1942) and in higher yield following *o*-aminoazotoluene (Andervont, 1950). More recently they have also been produced in mice after the subcutaneous administration at birth of 9,10-dimethyl-1,2-benzanthracene (Roe, Rowson and Salaman, 1961).

In an investigation of the effect of the subcutaneous administration of 9,10-dimethyl-1,2-benzanthracene (DMBA) given within 24 hours of birth to rats, primarily intended to study the effects on the haemopoietic system and lymphoma development, a considerable number of the animals developed haemangiomas. It is the purpose of this paper to describe the anatomical distribution and morphological appearances of these tumours, but it must be emphasised that they are only one aspect of a broad spectrum of changes, both neoplastic and non-neoplastic, observed in the experimental animals.

### MATERIALS AND METHODS

One hundred and thirty-four laboratory stock albino rats were injected subcutaneously with a 1·6 per cent solution of DMBA in olive oil. The needle was inserted just above the base of the tail, passed subcutaneously along the line of the vertebral column and 0·075 ml. of the solution (1·2 mg. DMBA) was deposited between the scapulae, this was to minimise seepage of oil from the inelastic tissues of the new born rat. When the animals were one month old they were weaned, sexed, and housed in groups of five in galvanised wire mesh cages. Rat cubes (Thompson Diet) and water were provided *ad libitum.*

### *Post mortem examination and histological methods*

At necropsy blocks from tissues showing pathological changes were fixed in 4 per cent formaldehyde-saline. As the haemangiomas when present were usually multiple it was impracticable to section all of them, and representative examples were fixed, cut at 5 $\mu$ and stained with Ehrlich's haematoxylin and eosin, Weigert's haematoxylin and Van Gieson, Lawson's modification of the Weigert-Sheridan elastic stain and Gomori's reticulin method. Additional stains used on occasions included the periodic-acid Schiff method and the Prussian-blue reaction for ferric iron.

### RESULTS

Survival of the experimental animals was surprisingly good and only 25 died during the first month of the experiment. Many of these had adrenal necrosis, and a few had to be killed because of skin ulceration over the site where the carcinogen was deposited. Growth and development of the remaining rats appeared unimpaired, and they were allowed to live their natural life span unless some complicating factor, e.g. the presence of a large sarcoma at the injection site, necessitated killing them. Some animals were cannibalised and complete post mortem examinations were made on 93 animals, of which 41 (44 per cent)

FIG. 1.—Incidence/time curves for the development of haemangiomas.

had haemangiomas. Nineteen females and 22 males had these tumours, the first example being found in a female 153 days after injection. The average tumour induction time was 319 days for females, and 358 days for males, and although the time of appearance of tumours in males lagged behind females, no major differences were observed and the incidence/time curves were roughly parallel (Fig. 1).

Haemangiomas, when present, were usually multiple and their anatomical distribution is given in Table I ; frequently animals had tumours in more than one of the sites listed. As can be seen the greater number (31) were found in the subcutaneous tissues and muscles. Twenty-seven were found in various internal organs, the commonest site being the uterine horns in females and the spleen in males. This natural division of tumours into a subcutaneous and soft tissue group, and an internal organ group, facilitates both macroscopic and microscopic description, hence they will be treated separately.

TABLE I.—*Anatomical Distribution of Haemangiomas*

| Site | Male | Female | Total |
|---|---|---|---|
| Subcutaneous and muscle | 16 | 15 | 31 |
| Mesenteric fat | 4 | 2 | 6 |
| Spleen | 7 | 1 | 8 |
| Uterine horns | — | 11 | 11 |
| Kidney | 1 | — | 1 |
| Brain | 1 | — | 1 |

### *Macroscopic Appearances*

### *Subcutaneous and soft tissue haemangiomas*

These were situated in any area of the body from scalp to legs, but only very rarely were they found in close proximity to the injection site. They frequently appeared as flattened disc-like lesions with poorly demarcated borders merging into adjacent tissues. Small lobulated, embossed, strawberry-like nodules bulging into the underlying soft tissues or muscles were also numerous. These were sharply circumscribed and could be readily dissected out (Fig. 2). Nearly all the haemangiomas had large vessels around the periphery, and vessels passing into, or leaving them. On cross-section some were solid, fleshy and compressible, most had small cystic areas filled with blood, others were completely cystic, collapsing with escape of fluid blood or blood clot when incised.

### *Visceral haemangiomas*

In the female the most frequent situation of haemangiomas involving internal organs was the uterine horn. They appeared as small red nodules attached to the serosal surface or else on the internal aspect growing into and expanding the lumen. They were usually found about midway along the length of the horn or near the ovary which itself was never the site of a tumour. With growth, large tumours were formed filling the lower abdomen, which were frequently bound to adjacent structures by dense adhesions making dissection difficult. Tumours of this size leaked blood, since bloody effusions within the peritoneal cavity were sometimes observed. Occasionally, a massive, spontaneous haemorrhage occurred causing the death of the animal.

In males the most frequent site of internal haemangiomas was the spleen. The earliest change noted was the development on the surface of multiple small, raised, circumscribed, plum-coloured lesions. At a later stage, a part, or the whole of the spleen was greatly enlarged with an irregular, variegated surface covered by adherent omentum and with adhesions to adjacent structures (Fig. 3). On cross section the spleen presented complex appearances with a multiplicity of cyst-like spaces containing fluid and clotted blood, the cut surface having a sponge-like quality. Areas of necrosis, fibrosis and focal calcification were sometimes present (Fig. 4). Again, these tumours were sometimes responsible for the death of the animal from intraperitoneal haemorrhage.

### *Microscopic Appearances*

For purposes of histological classification the tumours can be divided into a benign group which includes haemangiomas of capillary and cavernous type, a group with malignant potentiality which includes the much more complex and cellular haemangio-endotheliomas and haemangiopericytomas and a third small group of histologically malignant haemangiosarcomas (Table II). As the visceral

TABLE II.—*Histological Classification of Subcutaneous and Soft Tissue Haemangiomas*

| | | M. | F. | Total |
|---|---|---|---|---|
| Benign | Capillary and cavernous | 11 | 10 | 21 |
| Potentially malignant | Haemangio-endothelioma | 14 | 8 | 22 |
| | Haemangiopericytoma | 3 | — | 3 |
| Malignant | Haemangiosarcoma | 2 | 2 | 4 |

haemangiomas were all of the benign group and the subcutaneous and soft tissue haemangiomas contained examples of all histological types this anatomical division facilitates microscopic description.

*Subcutaneous and soft tissue haemangiomas*

As can be seen from Table II the tumours were divided between the benign and potentially malignant varieties with no sex difference in the benign group, but with a preponderance of the potentially malignant variety in males.

In males and females haemangiomas were found in mesenteric fat ; these had appearances essentially similar to some of those observed in the subcutaneous and soft tissues. They were frequently situated in close proximity to the pancreas and sometimes were adherent to spleen or other structures (Fig. 5). One animal had a haemangioma of the kidney, apparently arising in the renal pelvis and subsequently involving the renal parenchyma ; a further animal had a haemangioma of the brain which presented as progressive unilateral exophthalmos.

The benign haemangiomas were highly circumscribed lesions with no evidence of infiltration of the surrounding tissues which appeared to be pushed aside as the tumour expanded. They consisted of a collection of capillaries of varying calibre lined by a single layer of orderly endothelium (Fig. 6). The individual cells showed some variation in size and shape ; when the capillary was dilated the cells were elongated and flattened with darkly staining spindle-shaped nuclei, smaller capillaries tended to be lined by plumper cells with oval or spherical nuclei with either darkly staining chromatin material or else with a rather open vesicular appearance. Usually the capillary walls consisted of attenuated fibrous strands containing reticulin fibre (Fig. 7) but sometimes the walls were much thicker, composed of dense, relatively acellular collagen, with many reticulin fibres incorporated. The fibrous stroma might be so extensive as to give an impression of progressive fibrous obliteration of the lesion (Fig. 8). The calibre of the vessels varied, those in the centre of the lesion tending to be largest with smaller ones at the periphery. The usual gradual growth observed in these tumours appeared to be due to progressive dilatation of the smaller vessels and incorporation of peripheral vessels into the lesion associated with various complications such as thrombosis with subsequent organisation, and rupture of capillary walls. Thus these tumours were a mixture of capillary and cavernous haemangiomas similar in all respects to those observed in human pathology.

The potentially malignant haemangiomas were highly cellular lesions containing many more cells than required to line a collection of simple vascular channels (Fig. 9). Although the tumours were highly vascular, capillary structure was not always obvious and frequently required reticulin stains to reveal the underlying nature of the lesion. The cells and nuclei showed considerable variation in size and shape sometimes having rather bizarre appearances. They were closely arranged, several layers thick, oval or circular, sometimes lobulated in appearance, with either compact, darkly staining chromatin material or a network which was open and vesicular. Binucleated cells were sometimes seen. The stroma showed considerable variation ; in some there were thick bundles of collagen, sometimes fragmented, the appearance of which indicated that they had become incorporated into the tumour by its growth and infiltration ; in other tumours fibrous tissue was extremely scanty. In all tumours reticulin stains revealed a very rich reticulin network, constituting a complex system of arborising and anastomosing channels forming a basement membrane on which the cells were arranged, demonstrating beyond doubt the underlying vascular nature of the tumours (Fig. 10). From a study of the relationship between the cellular and reticulin components it was possible to divide these tumours into two types. In one, and by far the largest, the cells were arranged predominently within the reticulin network and the channels formed by it, thus corresponding to the haemangio-endothelioma of human pathology (Fig. 9 and 10). The other type, of which only three examples were found, corresponded to the haemangiopericytoma, in which the proliferating cells were arranged outside and in the interstices of a more complex reticulin network which failed to show the presence of such clear cut channels as in the haemangio-endothelioma (Fig. 11 and 12). Nevertheless apart from slightly more pleomorphic appearances, the basic cell types of these two varieties of tumour were similar. Both types of tumour showed pronounced infiltrative activity, unlike the simple variety described earlier ; invasion of the adjacent tissues including muscle (Fig. 13), fibrous tissue, fat, and breast tissue (Fig. 14), with incorporation of all these elements into the tumour were usual.

Around haemangiomas of all types there was usually a mild chronic inflammatory reaction in which mast cells were numerous ; mast cells were also present in the stroma of the tumours. Macrophages laden with haemosiderin were also plentiful. It was not unusual to find numbers of polymorphs within the lumen of the better formed capillaries.

Two males and 2 females had histologically undoubted malignant tumours of vascular or vasoformative tissue. These were all found in the subcutaneous tissues forming large ulcerating tumours with ill-defined borders, which on cut section had a soft, compressible, fleshy appearance. Microscopically they presented very pleomorphic appearances with variation in cell size, shape and staining reactions, but with a tendency for round cells with scanty cytoplasm or large plump cells with abundant cytoplasm to predominate. Tumour giant cells and aberrant mitotic figures were present (Fig. 15). In 3 of the tumours there were no obvious vascular channels although red cells were abundant ; the underlying nature only became apparent when reticulin was stained forming a complex system of channels on and around which the cells were arranged (Fig. 16). The stroma of these 3 tumours was myxomatous in appearance with very little, if any, collagen. In the fourth tumour, which was highly cellular, the cells showed a marked tendency to line cleft-like spaces and to form short cords and columns giving in some areas an overall papilliferous appearance with an extremely scanty fibrous stroma (Fig. 17). The reticulin pattern of this tumour showed the usual appearances with marked proliferation of cells into and around the channels formed by the reticulin fibres.

*Visceral haemangiomas*

In males the commonest organ to contain these was the spleen. Microscopically the first change noted was the development of a small group of widely dilated sinusoids usually situated immediately beneath the capsule. These sinusoids were stuffed with blood and the cells lining them were apparently derived from the existing sinusoidal wall (Fig. 18). This change was frequently multifocal involving several areas widely separated by splenic tissue with a normal sinusoidal pattern. The lesions developed by progressive dilatation and gradual incorporation of more involved sinusoids, eventually presenting the appearances of a cavernous haemangioma with enormous blood-filled spaces in the spleen (Fig. 19). The cells lining the cavernous spaces were all regularly arranged, only one layer thick without evidence of undue cellular activity. The walls were usually thin, consisting of acellular collagen which initially contained lymphoid elements of splenic tissue, but gradually these were lost and eventually it became impossible to recognise the tissue as spleen. Thrombosis with subsequent organisation of the thrombus and fibrosis and areas of focal calcification were also frequently observed. Despite the great enlargement of the spleen the capsule remained intact but sometimes showed a chronic lipogranulomatous inflammatory reaction spreading out into the adherent fat.

---

EXPLANATION OF PLATES

FIG. 2.—Dissection of rat to show multiple subcutaneous haemangiomas and a large cystic haemangioma in the neck (arrows).

FIG. 3.—Large haemangioma of spleen almost completely enveloped by adherent omental fat.

FIG. 4.—Cut surface of the splenic haemangioma seen in Fig. 3. Note sponge-like texture and organising blood clot.

FIG. 5.—Haemangioma of mesenteric fat adherent to pancreas and spleen (arrow).

FIG. 6.—Simple haemangioma. H. and E. ×27.

FIG. 7.—Reticulin pattern of a simple haemangiona. Reticulin × 78.

FIG. 8.—Simple haemangioma showing a well-developed fibrous stroma. H. and E. ×78.

FIG. 9.—Haemangio-endothelioma showing marked cellularity and fairly well-defined capillaries. H. and E. ×195.

FIG. 10.—Reticulin pattern of the haemangio-endothelioma in Fig. 9. Reticulin × 115.

FIG. 11.—Haemangiopericytoma showing pleomorphic appearances and obscure vascular channels. H. and E. ×195.

FIG. 12.—Reticulin pattern of haemangiopericytoma shown in Fig. 10. The vascular channels are rather ill-defined. Reticulin × 195.

FIG. 13.—Haemangio-endothelioma invading muscle. H. and E. ×78.

FIG. 14.—Haemangio-endothelioma invading breast tissues. H. and E. ×78.

FIG. 15.—Haemangiosarcoma showing cellular pleomorphism and myxoid stroma. H. and E. ×115.

FIG. 16.—Reticulin pattern of haemangiosarcoma illustrated in Fig. 15, demonstrating the underlying vascular nature of the tumour. Reticulin × 115.

FIG. 17.—Haemangiosarcoma with tendency for cells to line cleft-like spaces. H. and E. ×115.

FIG. 18.—An early stage in the development of a splenic haemangioma. Note several dilated sinusoids. H. and E. ×115.

FIG. 19.—Cavernous haemangioma of spleen. H. and E. ×78.

FIG. 20.—Haemangioma of mesentery of uterine horn. H. and E. ×12½.

FIG. 21.—Haemangioma involving brain. H. and E. ×3½.

FIG. 22.—Haemangio-endothelioma involving a mesenteric lymph node. Note tumour in periglandular fat. H. and E. ×18.

2

3

4

5

Howell.

6

7

8

9

10

11

12

13

14

15

16

17

18

19

20

21

22

Haemangiomas involving the uterine horns were all of the cavernous variety. Small congeries of vessels in the fibromuscular wall gradually dilated forming a small haemorrhagic nodule, gradually more vessels were incorporated and the lesion bulged into the lumen which frequently contained free blood or blood clot causing marked dilatation of the horn, the whole lesion being surrounded by attenuated fibromuscular tissue. They might also grow out to involve the serosal surface, leaving an intact, normal lumen and sometimes they appeared as pedunculated lesions within the fatty mesentery of the horn (Fig. 20). In none of these tumours was there any evidence of undue cellular activity, and no marked histological differences were observed between these tumours and the cavernous haemangiomas found in other sites, except that perhaps by virtue of their situation, they attained a larger size. Thrombosis, with organisation, and on occasions secondary infective changes were very common.

Haemangiomas of both the benign and the potentially malignant varieties involving mesenteric fat were found in males and females, but these did not differ in any way from the haemangiomas described in the subcutaneous tissues and muscles. The haemangioma of the kidney was capillary in type, and appeared to have arisen in the renal pelvis. The haemangioma of the brain, cavernous in type, arose in the choroid plexus and formed large blood filled spaces destroying much of one cerebral hemisphere (Fig. 21).

### DISCUSSION

The administration of DMBA at birth in this fashion obviously exerts a profound effect on the vasoformative tissues and perhaps, to a lesser extent, on mesenchyme as a whole. This effect is probably dependant upon the functional and structural immaturity of the tissues at the time of administration of the carcinogen, since at birth, tissues in general are in a labile state, final form and function still in progress.

The simple haemangiomas (capillary and cavernous) could attain a large size, but this growth was due to progressive dilatation of the involved vessels with increased blood flow through them associated with various other complications such as thrombosis and consequent opening up of other vascular channels. The progressive increase in size of some of these lesions could be correlated in certain instances with falling haemoglobin values in the peripheral blood. Histologically there was never any evidence of undue cellular activity, or aggressive behaviour and they were all highly circumscribed, and in some, as judged by the fibrous reaction there was evidence of spontaneous healing. Haemangiomas of this type were thus very similar in appearance and behaviour to their human counterpart, the latter being considered to arise as the result of tissue maldevelopment either *in utero* or in the perinatal period.

The potentially malignant haemangiomas, on the contrary, all showed infiltration of the surrounding tissues, and evidence of much cellular proliferation. From histological examination it was impossible to assess the probable biological behaviour of tumours of this type, but a strong impression was gained that at least some of them were biologically malignant. In a number of animals involvement of lymph nodes either in the abdomen or in the subcutaneous tissues were found. All these animals had haemangio-endotheliomas in other sites, similar in every way to those involving the node which therefore could be regarded as a metastasis. However, in every case the lesion in the node had burst through the capsule to involve the perinodal connective tissues (Fig. 22), therefore the nodes could equally have become involved by direct extension from a further primary haemangio-endothelioma, especially as the nodes were situated in areas known from the experimental results to be predisposed to the development of these tumours. Difficulty was also experienced in animals with haemangio-endotheliomas and small haemorrhagic lesions in the lungs. On section, the lung lesions appeared very like metastases, but haemorrhage obscured cellular details, and the lesions might equally be regarded as small areas of infarction.

The 4 haemangiosarcomas (one of which had metastasised to the lungs) formed large tumours in fairly close proximity to the site of deposition of the carcinogen between the scapulae, unlike the other types of vascular tumour which were never found in this situation. A variety of other sarcomas also developed at this site and many contained a wide diversity of neoplastic tissues, including vasoformative tissue, indicating that the tumours might be of mesenchymal origin (mesenchymomas) ; these have been excluded from the present paper. It thus appears probable that development of haemangiosarcomas is related to mesenchymal tissue damage at the site of carcinogen deposition and that the mechanism of development of this type of tumour may differ from that of the other varieties of haemangioma encountered.

The administration of DMBA at birth and its oncogenic effect bears some similarity to the effect of polyoma virus inoculated at birth. Both produce haemangiomas, although in the case of polyoma the yield is higher and the induction time is shorter (Kirsten *et al.*, 1962). The virus also produces renal and osteogenic sarcomas ; a few examples of renal sarcoma and a single osteogenic sarcoma were found in the DMBA injected rats all of whom had haemangiomas. The situation and yield of polyoma-induced haemangiomas is dependent upon the route of inoculation ; subcutaneous injection producing subcutaneous tumours; intraperitoneal and intravenous injection giving rise to a much wider distribution with a preponderance of cerebral haemangiomas, of which a single example was found in the DMBA injected rats. Nevertheless the interesting localisation of haemangiomas in the spleen and uterine horns is not produced by polyoma. From the similarities between the spectrum of polyoma- and DMBA-induced tumours it might be suggested that the effect of DMBA on the new born rat is to render overt a latent virus infection. There is no direct evidence on this point since formal viral studies were not undertaken. However, DMBA caused the development of lymphomas and leukaemia in a large number of animals. Some of these tumours were serially transplantable into new born rats when whole cell suspensions were used, but were not transplantable using cell free filtrates. The successfully transplanted animals died from lymphomatous lesions, but tumours of other types were never found. Thus these observations offer some indirect evidence against a viral factor in these experiments.

It must be stressed that spontaneous haemangiomas are very uncommon in the rat (Curtis, Bullock and Dunning, 1931) and have never been observed in the animals maintained in these laboratories, neither have the combinations of tumours found in these experimental animals been observed spontaneously. The experiments clearly show that in the new born rat the vascular system, and perhaps mesenchyme as a whole is extremely susceptible to carcinogenic stimuli, yielding a broad spectrum of interesting tumours not usually observed in the carcinogen treated adult animal of which haemangiomas are but one example.

SUMMARY

Experiments are described which show that haemangiomas are produced in many rats following the injection at birth of a solution of DMBA in olive oil. The anatomical distribution and microscopic appearances of these tumours are described in detail. A certain similarity between the effects of polyoma virus inoculated into new born rats and DMBA injected at birth is noted and briefly discussed.

### REFERENCES

Andervont, H. B.—(1950) *J. nat. Cancer Inst.*, **10**, 927.
Curtis, M. R., Bullock, F. D. and Dunning, W. F. A.—(1931) *Amer. J. Cancer*, **15**, 67.
Kirsten, W. H., Anderson, D. G., Platz, C. E. and Crowell, E. B.—(1962) *Cancer Res.*, **22**, 484.
Roe, F. J. C., Rowson, K. E. K. and Salaman, M. H.—(1961) *Brit. J. Cancer*, **15**, 515.
White, J. and Stewart, H. L.—(1942) *J. nat. Cancer Inst.*, **3**, 331.